# How Would the World Look if It Looked as if It were Encoded as an Intertwined Set of Probability Density Distributions?

**DOI:** 10.3389/fpsyg.2012.00419

**Published:** 2012-10-18

**Authors:** Michael Madary

**Affiliations:** ^1^Johannes Gutenberg-Universität MainzMainz, Germany

In the target article, Andy Clark addresses the question of how a probabilistic predictive coding model of the mind relates to our personal level mental lives. This question, he suggests, is “potentially the most important” (MS46). The question is important indeed, but Clark’s answer fails to capitalize on another possible advantage of this approach. Clark suggests that there is a disconnect between the way the world appears to us, on one hand, and the way that it is represented in the brain, on the other. He deals with this disconnect by limiting the scope of the theory, by pointing out that he is discussing a theory of how brains encode and process information, not a theory about how things seem to organisms with such brains. The shortcoming of this strategy is that there may not be a disconnect to begin with. That is, perhaps the world does appear to us as if it were “encoded as an intertwined set of probability density distributions” (MS47). If such is the case, then explanations which appeal to a probabilistic predictive model gain even more scope and power. Here I will offer a sketch of both *a priori* and empirical support for my claim.

One emerging theme in the philosophical literature is that perception involves implicit anticipation of the way appearances change (Noë, 2004; Siegel, 2006; Madary, 2012). Here is an outline of the motivation behind this view. The fact that we are embodied perceivers entails that we are always limited to a single perspective on the world at one time. Perception faces the task of representing properties despite only having access to a single appearance of those properties at any time. A straightforward way to handle this task is to represent properties by implicitly anticipating how appearances of those properties will change as we move. As those anticipations are fulfilled, we gain more evidence for our representation. It would be natural, following Clark and others, to account for perceptual anticipations in a probabilistic manner. If an object looks like a tree from one perspective, it is probable that it will continue to look like a tree from other perspectives. This kind of perceptual anticipation is not usually the center of our attention, but, crucially, neither is it hidden away in sub-personal code. We can plainly observe the changing appearances of static properties and we are surprised when appearances do not change as they should.

This point raises the tricky question of the relationship between personal level surprise, on one hand, and the sub-personal prediction error, known as “surprisal,” on the other (MS5). Personal level surprise is an experience with which we are all familiar, and sub-personal surprisal is a key component of the approach that Clark is exploring. The relationship between the two strikes me as an important unsolved issue. In the target article, Clark offers a reconciliation for the apparent disconnect between personal level surprise and sub-personal surprisal (MS46). Elsewhere, though, Clark concedes that “Although the psychological notion of surprise is distinct, events with high surprisal are generally surprising” (Friston et al., 2012, p. 1). I suggest that an account of when and why surprisal (sub-personal) is surprising (personal) will be key for addressing the larger question of the relationship between sub-personal level processing and personal level phenomenology. Leaving this difficult issue aside, now consider the psychological evidence that suggests vision is indeterminate, evidence which may fit nicely with the suggestion that visual processing uses probabilistic coding.

Two lines of empirical evidence show that visual perception is an ongoing process which involves repeated sampling of the environment. Such a structure to visual perception fits naturally with a probabilistic interpretation: we take repeated samples in order to update our best estimate of the way the world is. First, our experience of the visual periphery is highly indeterminate (Cohen and Dennett, 2011). We are able to experience parts of the world in a determinate manner through our ability continuously to gain different perspectives through action (Findlay and Gilchrist, 2003). Second, both inattentional blindness (Mack and Rock, 1998) and change blindness (Rensink et al., 1997) paradigms suggest that our experience of the world can lack basic details. Both of these lines of evidence fit nicely with an understanding of visual experience as probabilistic. The indeterminacy of the visual periphery can be interpreted as a probabilistic representational format. Similarly, the missing details as revealed in inattentional and change blindness experiments reveal that our generative models are more successful with the gist of a visual scene and offer only vague estimates about the details.

In short, both the general *a priori* structure of perception and recent evidence in perceptual psychology converge on the theme that visual experience involves indeterminate implicit anticipations. This theme fits quite well with the suggestion, explored by Clark, that the brain actively predicts sensory inputs in a probabilistic manner. Far from the conflict that Clark supposes, the predictive generative model of perceptual processing might complement our best account of the phenomenology of vision.

## References

[B1] Cohen M. Dennett D. (2011). Consciousness cannot be separated from function. Trends Cogn. Sci. (Regul. Ed.) 15, 358–364 10.1016/j.tics.2011.10.004 21807333

[B2] Findlay J. M. Gilchrist I. (2003). Active Vision: The Psychology of Looking and Seeing. London: Oxford University Press

[B3] Friston K. Thornton C. Clark A. (2012). Free-energy minimization and the dark-room problem. Front. Psychol. 3:130 10.3389/fpsyg.2012.00130 22586414 PMC3347222

[B4] Mack A. Rock I. (1998). Inattentional Blindness. Cambridge: MIT Press

[B5] Madary M. (2012). Anticipation and variation in visual content. Philos. Stud. 10.1007/s11098-012-9926-3

[B6] Noë A. (2004). Action in Perception. Cambridge: MIT Press

[B7] Rensink R. A. O’Regan J. K. Clark J. J. (1997). To see or not to see: the need for attention to perceive changes in scenes. Psychol. Sci. 8, 368–373 10.1111/j.1467-9280.1997.tb00427.x

[B8] Siegel S. (2006). Subject and object in the contents of visual experience. Philos. Rev. 115, 355–388 10.1215/00318108-2006-003

